# Will early detection of non-axillary sentinel nodes affect treatment decisions?

**DOI:** 10.1038/sj.bjc.6600557

**Published:** 2002-09-23

**Authors:** F Wärnberg, N Bundred

**Affiliations:** Department of Surgery, Uppsala University Hospital, Sweden; Department of Surgery, South Manchester University Hospital, UK

**Keywords:** breast cancer, non-axillary sentinel node, treatment

## Abstract

Axillary lymph node involvement is the best prognostic factor for breast cancer survival. Staging breast cancers by axillary dissection remains standard management and is part of the UK national guidelines for breast cancer treatment. In the presence of involved axillary lymph nodes best treatment has been shown to be axillary clearance (Fentiman and Mansell, 1991), but clearly for women whose nodes are uninvolved avoidance of morbidity is optimal and this will be achieved by minimal dissection of the axilla. Thus, for node-negative women the introduction of the sentinel node biopsy technique may revolutionise the approach to the axilla. These will be women with mammographic screen detected small well and moderately differentiated tumours (Hadjiloucas and Bundred, 2000). The impact of sentinel node biopsy in women who have symptomatic large tumours is unproven, and around half of these women will require a second procedure to clear their axilla or radiotherapy as treatment. Even for those women found to have involved sentinel lymph nodes the ability to use early systemic chemotherapy followed by axillary clearance or radiotherapy may provide long-term survival gains. Sentinel node biopsy should not, however, become routine practice until randomised controlled trials have proven its benefit and safety in reducing morbidity. Several randomised controlled trials (including ALMANAC) are currently underway.

*British Journal of Cancer* (2002) **87**, 691–693. doi:10.1038/sj.bjc.6600557 www.bjcancer.com

© 2002 Cancer Research UK

## 

Sentinel node lymphoscintigraphy leads to the visualisation of hot radioactive nodes in sites other than the axilla in about 13% of all cases (range 2–35% in series using different injection techniques) (Cserni and Szekeres, 2001). Tanis and co-workers in this issue of the Journal report the detection of non-axillary sentinel nodes and its impact on treatment. Earlier studies where internal mammary (IM) lymph node dissection has been performed have shown IM node involvement in about 23% of breast cancer patients (Cserni and Szekeres, 2001). In the study by Tanis *et al* (this issue) the proportion of detected non-axillary node metastases was about 5% among all patients, which is similar to findings in other studies where lymphoscintigraphy has been used (Cserni and Szekeres, 2001; Dupont *et al*, 2001). Only patients with a visualised non-axillary sentinel node were biopsied but this low proportion of metastases might also reflect that breast cancer is detected at an earlier stage today. The proportion of involved nodes was 20–25% among those patients who underwent an IM or other non-axillary sentinel node biopsy.

Centrally and medially located tumours have been reported to have a higher proportion of IM lymph node metastases compared to lateral tumours (Manji, 1982). However, in the overview of series undergoing extended radical mastectomy (Cserni and Szekeres, 2001) the range of IM lymph node involvement was 13.3–35.3% in lateral tumours and 19.5–32.6% in central and medial tumours. Tumour location alone is therefore not a reliable indicator of risk for IM lymph node metastases (Urban and Marjani, 1971; Donegan, 1977; Noguchi *et al*, 1998, 2000; Sugg *et al*, 2000; Cserni and Szekeres, 2001; Dupont *et al*, 2001).

The detection of an involved sentinel node in non-axillary sites (predominantly IM nodes) is not necessarily of prognostic value or predictive of survival. The prognostic value of IM lymph node involvement alone is similar to the value of axillary lymph node involvement (Noguchi *et al*, 1993; Veronesi *et al*, 1999). However, the importance of finding a non-axillary sentinel node will mainly be dependent on it being the only involved node which occurs in less than 10% of cases where the IM nodes are involved (Donegan, 1977; Jansen *et al*, 2000). A small proportion of axillary node negative patients have IM node metastases but today, we are not able to predict which patients these are. The value of finding these metastases remains to be determined both regarding prognosis and affect on treatment decisions.

The detection of IM nodes or other non-axillary sentinel nodes is largely dependent on the method of injection of isotope and the use of lymphoscintigraphy to detect involved nodes. A peri-tumoural injection technique visualises IM lymph nodes in about 15–30% of women, whereas subdermal or subareolar injection of isotope does not seem to identify IM nodes (Kett *et al*, 1993; Borgstein *et al*, 1997, 2000; Roumen *et al*, 1999; Cserni and Szekeres, 2001; Shen *et al*, 2001; Tanis *et al*, 2001). The mammary gland and the overlying skin clearly show a common lymphatic pathway to the axilla and the same axillary sentinel node in most cases (Dupont *et al*, 2001), but they do not appear to both drain to the IM chain. The kinetics of different traces and the timing of the injection before surgery are also issues that influence the detection and successful localisation of an IM node, e.g. lymph drains more slowly after a peri-tumoural injection compared to after an intra-dermal sub-areolar injection. Furthermore, the preoperative intra-dermal injection of colloid blue dye quickly disappears and makes it more difficult to find very small IM nodes compared with an intra-tumoural or peri-tumoural injection especially if the IM node dissection is performed at the end of an operation including axillary dissection and primary tumour excision. The use of blue dye alone should be discouraged as it gives sub-optimal imaging for non-axillary sentinel nodes.

Although some authors have argued we should abandon lymphoscintigraphy (Mcintosh and Purushotham, 1998) because most axillary sentinel nodes can be detected at operation by gamma-probe, its discontinuation would preclude the detection of IM nodes or non-axillary sentinel nodes. Thus, lymphoscintigraphy is particularly crucial for medial tumours in the breast in young women less than 70 years of age where chemotherapy may be indicated if a positive node is found in a non-axillary sentinel node but not in the axilla. However, it must also be recognised the treatment decisions are based on tumour characteristics (Goldhirsch *et al*, 1995). Large size, high grade, hormone receptor negativity and other prognostic factors generally indicate the need for chemotherapy regardless of node status at present. The non-axillary node status will still add information on prognosis for these women but will not affect treatment decisions.

In the study by Tanis *et al* (this issue) patient management changed in 17% of patients with visualised non-axillary sentinel nodes. However, this may be an overestimation of the importance of non-axillary sentinel node biopsies. As shown in various randomised studies comparing Halsted mastectomy with extended mastectomy, the IM lymph node dissection by itself has no survival benefit (Lacour *et al*, 1976; Donegan, 1977; Veronesi *et al*, 1985, 1999). Another question to be answered is the role of radiotherapy as treatment to the IM chain. Thus far radiotherapy has not provided any survival advantage but the issue is still under study and a large European trial EORTC 22922 is investigating the effects of irradiation on the IM and medial supraclavicular lymph nodes in terms of loco-regional control and survival (Lievens *et al*, 2001). The routine practice in the department of Tanis *et al* (this issue) was to irradiate the parasternal area if axillary nodes were involved. In a large proportion of the cases where management ‘changed’ the use of radiotherapy was decided on the basis of the IM node status; a therapy that is unproven. Furthermore, eight out of 11 patients with isolated non-axillary lymph node metastases had tumours larger than 1 cm and would therefore have been offered adjuvant hormonal treatment according to many treatment guidelines anyway. The routine use of immunohistochemistry in addition to H&E staining to detect nodal micro-metastases is not accepted practice in many centres and additionally, the prognostic value of metastases lesser than 2 mm in size is not proven (Yarbro *et al*, 1999). Thus the value of treating such findings is unknown and itself justifies a clinical trial comparing adjuvant treatment with no adjuvant treatment for women with nodal micro-metastases.

The benefit of IM sentinel node biopsy has to be weighed against the possible morbidity caused by surgery, as women who undergo breast-conserving surgery will need a further medial incision over the sternum to biopsy the IM node. The lack of interest in the IM node means that many surgeons today have no experience of the procedure nor have they had to deal with the complications of the procedure such as haemo- or pneumothorax (Bembenek and Schlag, 2000). However, a few studies have shown that non-axillary sentinel node biopsy can be performed with few side effects and a relatively high success ratio (Jansen *et al*, 2000; Noguchi *et al*, 2000; Dupont *et al*, 2001) although this has to be confirmed in other centres and countries.

The value of detecting non-axillary lymph nodes by lymphoscintigraphy potentially improves the rationale for individualising optimal treatment strategy. Biopsy of an IM lymph node is necessary if lymphoscintigraphy indicates it is the sentinel node, though, it remains inappropriate to biopsy an IM node if it is not blue or hot on lymphoscintigraphy as IM node biopsy is not routine practice. First and foremost, data from randomised trials are required to demonstrate that sentinel node biopsy is reducing morbidity in axillary surgery. Once such data is available we will be able to determine if the identification of non-axillary sentinel lymph nodes contributes to treatment decisions significantly.
